# *Borrelia burgdorferi* BmpA-BBK32 and BmpA-BBA64: New Recombinant Chimeric Proteins with Potential Diagnostic Value

**DOI:** 10.3390/pathogens10060767

**Published:** 2021-06-18

**Authors:** Weronika Grąźlewska, Bartłomiej Ferra, Monika Rudzińska, Lucyna Holec-Gąsior

**Affiliations:** 1 Department of Molecular Biotechnology and Microbiology, Faculty of Chemistry, Gdańsk University of Technology, 80-233 Gdańsk, Poland; wergrazl@student.pg.edu.pl (W.G.); barferra@pg.edu.pl (B.F.); 2 Department of Tropical Medicine and Epidemiology, Institute of Maritime and Tropical Medicine, Medical University of Gdańsk, 81-519 Gdynia, Poland; monika.rudzinska@gumed.edu.pl

**Keywords:** *Borrelia burgdorferi* sensu lato, chimeric proteins, Lyme disease, serodiagnosis

## Abstract

Currently, the diagnosis of Lyme disease is based mostly on two-tiered serologic testing. In the new generation of immunoenzymatic assays, antigens comprise whole-cell lysates of members of the *Borrelia burgdorferi* sensu lato (s.l.) species complex, with the addition of selected recombinant proteins. Due to the high diversity of members of the *B. burgdorferi* s.l. genospecies and the low degree of conservation among the amino acid sequences of their proteins, serodiagnostic methods currently in use are not sufficient for the correct diagnosis of borreliosis. Two divalent chimeric proteins (BmpA-BBK32 and BmpA-BBA64) were expressed in *Escherichia coli*. Following purification by one-step metal-affinity chromatography, preparations were obtained containing milligram levels of chimeric protein exhibiting electrophoretic purity in excess of 98%. Reactivity of the new chimeric proteins with specific human IgG antibodies was preliminarily determined by Western blot. For this purpose, 20 negative sera and 20 positive sera was used. The new chimeric proteins were highly reactive with IgG antibodies contained in the serum of patients suffering from borreliosis. Moreover, no immunoreactivity of chimeric proteins was observed with antibodies in the sera of healthy people. These promising results suggest that new chimeric proteins have the potential to discriminate between positive and negative sera.

## 1. Introduction

Lyme disease (LD) is the most common tick-borne human disease in the Northern Hemisphere. The infection is caused by spirochetes belonging to the *Borrelia burgdorferi* sensu lato (s.l.) complex. Currently, there are about 20 genospecies in this group, yet new members are still being discovered. At least five genospecies making up *B. burgdorferi* s.l. are the causative agents of LD in Europe: *Borrelia afzelii, Borrelia garinii, Borrelia burgdorferi* sensu stricto (s.s.), *Borrelia bavariensis, Borrelia spielmanii* [1,2]. Until recently, it was considered that the only causative agent of LD in North America was *B. burgdorferi* s.s.; however, recent reports suggest that *Borrelia bisettii* and *Borrelia mayonii* may also cause LD in the USA and Canada [3,4].

The genomes of members of the *B. burgdorferi* s.l. complex are very unique relative to most other bacteria. They consist of a linear chromosome and at least 21 plasmids, both linear and circular. Among such a large number of plasmids, it seems that only two (lp54 and cp26) are present in all isolates of *B. burgdorferi* s.l. This means that differences in the genomes of individual *B. burgdorferi* s.l. isolates can be significant [5,6,7]. The chromosome consists mostly of housekeeping genes, and its nucleotide sequence is fairly well conserved. On the plasmids are the majority of genes encoding lipoproteins responsible for the virulence of *B. burgdorferi* s.l. Many of the plasmids are easily lost during in vitro cultivation, and therefore, laboratory culture of fully virulent strains of *B. burgdorferi* s.l. is extremely difficult [5,8,9]. 

The enzootic life cycle of *B. burgdorferi* s.l. is very complex, and therefore, different genes are expressed at different stages of the life cycle. Especially great changes in the antigenic profile take place during the migration of *B. burgdorferi* s.l. from the tick midgut and salivary glands into mammalian tissue [10]. Changes in the expression of individual genes occur in response to variations in the temperature and pH value of the pathogen’s environment. This means that the antigenic composition of *B. burgdorferi* s.l. differs significantly between individual isolates based on the prevailing conditions. Many of the proteins whose production depends on environmental conditions are expressed on the surface and are responsible for the interaction of bacteria with the host tissues [10,11].

*B. burgdorferi* s.l. surface proteins are divided into two main groups: lipoproteins, which are anchored to the outer membrane via lipid moieties at the N-terminal end, and integral outer membrane proteins (OMPs), which contain transmembrane-spanning domains. OMPs provide critical physiological functions for the bacterial cell, including nutrient acquisition, protein transport and assembly, antibiotic resistance, and cellular adhesion. OMPs are mainly encoded by genes located on the chromosome, and therefore, their amino acid sequences are fairly well conserved. OMPs are not particularly immunogenic, so despite their highly conserved sequences, they are not widely used in the serodiagnosis of Lyme disease [12]. On the other hand, lipoproteins are highly immunogenic and are often used in serodiagnosis tests. Unfortunately, they are mainly encoded by genes located on plasmids, and therefore, they are characterized by a low level of amino acid sequence conservation between individual *B. burgdorferi* s.l. isolates [10,13,14,15]. Surface lipoproteins mediate transition through the enzootic cycle, so their expression pattern undergoes clear changes during the life of the spirochete. They also play an important role in virulence and in host-pathogen interactions. It had been proven that isolates unable to express some of the major lipoproteins (e.g. OspC, DbpA, and BBA64) either decreased, or completely lost, their virulence. Additionally, the strong immunogenicity and high amino acid sequence variability of these lipoproteins means that they play a role in avoiding the host’s immune response [9,11,16,17].

Because the clinical manifestations of Lyme disease are very diverse and non-specific, diagnosis of borreliosis usually requires confirmation by means of a laboratory diagnostic test. Direct pathogen detection methods are not routinely used. Molecular methods support serodiagnosis in the early stage of Lyme disease (before antibody response occurs) and in the diagnosis of patients suffering from dysfunction of the immune system [1,18]. Bacterial culture is the gold standard for microbiological diagnosis. However, *B. burgdorferi* s.l. requires complex media for growth, and the process is very time-consuming; therefore, this method is not widely used in recognition of borreliosis [1,19].

The more common current method for laboratory diagnosis of Lyme disease is the detection of anti-*Borrelia* antibodies in serum samples. A two-step testing strategy for the serodiagnosis of Lyme disease has been recommended both in the USA and in Europe. First, a sensitive enzyme-linked immunosorbent assay (ELISA) is used as a screening test; then, positive or borderline results are confirmed by a more specific Western blot (WB) [20,21,22,23]. Commercially available assays for the detection of specific immunoglobulins use either native whole-cell antigens, purified antigens (flagellar components), or whole-cell antigens combined with recombinant proteins. Unfortunately, the available serological tests are often unsuitable or insufficient for the diagnosis due to the large antigenic diversity of the *B. burgdorferi* s.l. group and a high level of cross-reactivity. In Europe, serological testing for borreliosis must consider the heterogeneity of causative agents. 

An additional drawback may be a problem with the standardization of ELISA and WB assays based on whole-cell lysates. It can be difficult to obtain cell lysates that contain all of the most immunogenic antigens, since the genes encoding some of them are only expressed in vivo. In addition, the ability to produce some proteins is lost with the plasmids encoding them, as a result of repeated passaging [1,9,11,24]. Research is underway to construct novel antigens using genetic engineering methods. These recombinant and chimeric proteins can be characterized by both high reactivity with specific antibodies and reduction of the risk of cross-reactions.

The aim of this study was the construction and production of two new chimeric proteins (BmpA-BBA64 and BmpA-BBK32) and preliminary determination of their reactivity with human immunoglobulin G (IgG). The antigenicity of chimeric proteins against human serum samples was evaluated by WB analysis. Our results suggest that these chimeric proteins might be useful for the detection of specific antibodies against *B. burgdorferi* s.l.

## 2. Results

### 2.1. Amino Acid Sequence Analysis

In order to select suitable antigen fragments for the construction of chimeric proteins, the amino acid sequences of known surface lipoproteins of five *B. burgdorferi* s.l. genospecies were analyzed. Based on results obtained in aligned amino acid sequences, it was decided to construct two chimeric proteins: the first (designated BmpA-BBA64) containing a fragment of the proteins BmpA (residues 58–339) and BBA64 (residues 171–302) and the second (designated BmpA-BBK32) containing a fragment of the proteins BmpA (residues 58–339) and BBK32 (residues 204–354). Amino acid sequence alignments for selected lipoproteins are available in the Supplementary Materials.

### 2.2. Expression and Purification of Chimeric Proteins BmpA-BBA64 and BmpA-BBK32

BmpA-BBA64 and BmpA-BBK32 were expressed in *Escherichia coli* BL21(DE3)pLysS as recombinant chimeric proteins with calculated molecular masses of 51 and 53 kDa, respectively. Both contained His_6_-tag domains at the N- and C-termini for purification of recombinant proteins by one-step metal-affinity chromatography. Preparations were obtained with electrophoretic purity greater than 98% (results not shown). This prokaryotic expression system produced approximately 32 and 29 mg of purified BmpA-BBA64 and BmpA-BBK32 proteins per liter of culture, respectively.

### 2.3. Western Blotting

**Figure 1:**
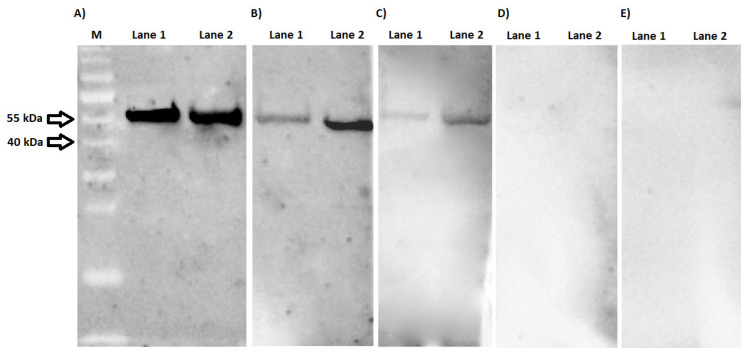
Western blot analysis. Purified BmpA-BBK32 (lane 1) and BmpA-BBA64 (lane 2) tested with anti-*Borrelia* antibodies from human serum samples. IgG levels in the positive serum samples expressed in relative units/ml (RU/mL): (**A**) 203 RU/mL, (**B**) 98 RU/mL, (**C**) 59 RU/mL. Membranes (**D**,**E**) were incubated with serum samples from seronegative patients. M—molecular mass marker (#26619; Thermo Scientific, Waltham, MA, USA).

Preliminary determination of reactivity of chimeric proteins with IgG specific antibodies was conducted with a of 40 known human serum samples (20 positive and 20 negative). IgG antibodies present in all 20 serum samples from patients with borreliosis efficiently recognized the BmpA-BBA64 and BmpA-BBK32 (Table S1 in Supplementary Materials). Figure 1 shows representative results for three positive and two negative sera. Signal intensity in the WB was related to the antibody titer in the human serum. BmpA-BBA64 appears to be more efficiently recognized by specific anti-*Borrelia* antibodies than BmpA-BBK32. Furthermore, no immunoreactivity of either chimeric protein was observed with serum samples from healthy patients (membranes D–E).

## 3. Discussion

The most common diagnostic method of Lyme disease is a 2-tiered testing strategy using ELISA and WB assays. The latest generation of commercial serological tests (both ELISA and WB) are based on whole-cell lysates with the addition of recombinant proteins (the most common being VlsE, DbpA, and OspC). There are several disadvantages to using whole-cell lysate as a source of antigens in serological tests, the main ones being high production cost, time requirements, inconsistent quality, batch-to-batch variation, and exposure of staff to harmful living pathogens, as well as the relatively high probability of cross-reactions [1,19,25,26]. *B. burgdorferi* s.l. proteins may demonstrate cross-reactivity with antigens of such pathogens as *Borrelia hermsii*, *Treponema pallidum*, *Treponema phagedenis*, *Yersinia enterocolitica*, *Yersinia pseudotuberculosis*, *Leptospira interrogans, Neisseria meningitidis, Haemophilus influenzae, Campylobacter jejuni, Listeria monocytogenes, Pseudomonas aeruginosa, E. coli, Salmonella enterica serovar Typhimurium, Shigella flexneri, Legionella micdadei*, and Epstein–Barr virus. In addition, patients suffering from inflammatory diseases such as periodontal disease and rheumatoid arthritis may also have antibodies that react nonspecifically with *B. burgdorferi* s.l. antigens [1,25,26,27]. However, the greatest problem in the serodiagnosis of Lyme disease seems to be the exceptionally high antigenic diversity of *B. burgdorferi* s.l. itself. Therefore, antibodies specific for one genospecies may not recognize epitopes from another member of the *B. burgdorferi* s.l. complex. This problem applies in particular to Europe, where five *B. burgdorferi* s.l. genospecies have been confirmed as causative agents of LD in humans. In addition, the complex life cycle of *B. burgdorferi* s.l. and the large number of plasmids (which are easily lost) mean that different antigens are produced at each stage of the life of the bacteria, which makes the selection of reference candidates for serodiagnostic assays extremely difficult [1,28,29,30].

For these reasons, intensive research is currently underway to develop new diagnostic tools for more efficient and more specific serodiagnosis of borreliosis. The use of molecular biology techniques makes it possible to produce a larger quantity of recombinant proteins for the serodiagnosis of LD with lower production costs. Moreover, the use of recombinant proteins in diagnostic kits instead of whole-cell lysates may reduce kit-to-kit variations in quality, enabling the development of a more specific and standardized serological assay. It has been demonstrated that recombinant proteins such VlsE, DbpA, OspC, BBK32, BmpA, and BBA64 are suitable tools in making a specific diagnosis of Lyme disease [31,32,33,34,35]. Unfortunately, one single protein may not be sufficient to create a high-sensitivity serodiagnostic test, as it may have too few epitopes recognized by specific antibodies to generate an intense signal in an immunoenzymatic assay. One solution to the problem of insufficient sensitivity may be to use mixtures of several recombinant proteins instead of a single one. This approach has been shown to increase sensitivity in immunoenzymatic tests for diseases caused by other pathogens [36,37,38,39,40]. However, the biotechnological production and purification of a large number of different proteins can be expensive and time-consuming. Furthermore, standardization of tests based on several types of proteins can also be problematic. The solution to these problems may be to use chimeric proteins. These recombinant proteins combine regions of high immunogenicity derived from two (or more) different antigens into one, single polypeptide. Chimeric products are better at detecting the presence of specific antibodies than either single proteins or mixtures, as demonstrated in, for example, the diagnosis of toxoplasmosis or Chagas disease [41,42,43,44,45,46,47]. 

As chimeric antigens have been proven effective in the serodiagnosis of other diseases, they may also help to resolve problems in the recognition of Lyme disease caused by the presence of multiple different genospecies. Bioinformatic analysis facilitates the identification of immunogenic amino acid sequences conserved across all *B. burgdorferi* s.l. genospecies [48,49,50,51]. Furthermore, the use of protein fragments (rather than entire polypeptides) can lead to significant reduction in cross-reactions. 

Until now, there have been only a few studies demonstrating the usefulness of chimeric proteins in detecting specific antibodies against *B. burgdorferi* s.l. in human sera [52,53,54]. In 2000, Gomes-Solecki et al. constructed 17 chimeric proteins (both divalent and trivalent) containing different fragments of five antigens: OspA, OspB, OspC, flagellin (Fla or p41), and p93 [52]. However, only two demonstrated high reactivity with specific antibodies (OspA-p93 (97 kDa) and OspB-OspC-Fla (64 kDa)). Gomes-Solecki et al. showed that ELISA assays based on these chimeric proteins can detect antibodies significantly better in early Lyme disease than commercial whole-cell *B. burgdorferi* assays, and they demonstrated equivalent sensitivity when tested with late LD sera [52]. The abovementioned chimeric proteins were also mixed with the two OspC proteins (from *B. garinii* and *B. afzelii*) and used in a rapid immunochromatography test. The sensitivity and specificity of this assay were also found to be equivalent to the commercial ELISA assay. The main advantages of this test were its speed and the use of whole blood (from a finger). This means that a screening assay could be performed during a short visit to the doctor. [53]. Another chimeric protein described in the literature, DbpA/C6, is composed of full-length DbpA linked with the short peptide derived from invariable region 6 of the VlsE (peptide C6). Results of this test suggest that this antigen, combined with OspC, could be used in a simple 1-tier ELISA that is better at detecting anti-*Borrelia* antibodies in sera from patients with acute LD than the standard 2-tier test method [54].

In this current work, a prokaryotic expression system has been constructed for the first time to produce two new divalent chimeric proteins (BmpA-BBA64 and BmpA-BBK32), and estimated their reactivity with specific anti-*Borrelia* antibodies. For the construction of chimeric proteins, fragments of the BmpA, BBA64, and BBK32 antigens were used. All three antigens are lipoproteins anchored in the outer membrane and play a prominent role in the spread and maintenance of *B. burgdorferi* s.l. in the mammalian host [12,55,56]. BBA64 and BBK32 are encoded by genes located on plasmids lp54 and lp36, respectively, whereas the *bmpA* gene is located on the chromosome; therefore, the BmpA amino acid sequence is exceptionally well conserved [12,57]. Furthermore, these antigens are also characterized by high immunogenicity, as confirmed by numerous reports [31,32,35,58,59].

Both novel chimeric proteins (BmpA-BBA64 and BmpA-BBK32) were comprised of the same fragment of the BmpA antigen at their N-terminal end. The C-terminus contained amino acid sequences derived from either the BBA64 or BBK32 antigen. The prokaryotic expression system allowed for easy and efficient chimeric protein production. Following purification, preparations were obtained with milligram levels of chimeric protein content and electrophoretic purity greater than 98%.

The preliminary reactivity of the purified chimeric proteins was evaluated by WB analysis with sera from healthy individuals and patients suffering from Lyme disease. For the test, 40 sera with known antibody titers were used. Both chimeric proteins were recognized by antibodies contained in positive serum samples. It appears that BmpA-BBK32 is less reactive with specific anti-*Borrelia* antibodies. This is particularly visible for sera with antibody level below 100 RU/mL (Figure 1B,C). There may be two reasons for this: firstly, the *bbk32* gene is located on plasmid lp36, which is not present in all *B. burgdorferi* s.l. isolates; secondly, the fragment of BBK32 antigen was the most diverse of the three selected for construction of chimeric proteins [5,8]. Additionally, the signal intensity for both chimeric proteins is proportionally dependent on IgG titer. This may indicate that the epitope/paratope interactions occurring here are highly specific. This assumption is supported by the fact that no protein was recognized by antibodies present in sera defined as negative. However, too few sera have been tested to draw any further conclusions from this observation.

Nevertheless, these results are promising and show that these novel chimeric proteins are recognized by specific antibodies, suggesting that they have the potential for discrimination between positive and negative sera. We only had basic information about the sera used in this study. Probably all of them were obtained from individuals living in a very close area. Therefore, we cannot exclude that the antibodies contained in them recognized antigens only of one or two *B. burgdorferi* s.l. genospecies. In order to verify whether the chimeric proteins BmpA-BBK32 and BmpA-BBA64 are reactive with antibodies directed against a wide range of genospecies, it is necessary to conduct further studies using a group of well-defined sera. For example, using sera from animals experimentally infected with different *B. burgdorferi* s.l. genospecies would allow us to determine how the immunoreactivity of our chimeric proteins differs depending on the causative agent of Lyme disease. There is also the possibility that not all *B. burgdorferi* s.l. genospecies have been found. It is still under debate as to whether *Borrelia bissettii*, *Borrelia lusitaniae,* and *Borrelia valaisiana* are pathogenic to humans. It may turn out that the antigen fragments used by us are not conserved in these genospecies, which would reduce the diagnostic usefulness of the constructed chimeric proteins. For better estimations of their reactivity with anti-*Borrelia* antibodies, it will be necessary to carry out further studies on a larger pool of sera using WB as well as ELISA assays. A key finding will be to determine the reactivity of constructed chimeric proteins with sera containing specific antibodies against the epitopes of phylogenetically related microorganisms that may be the source(s) of cross-reactivity. 

The development of the diagnosis of infectious disease is a complex task involving the search for new sources of suitable antigens. Chimeric proteins may be a new generation of tools for the diagnosis of borreliosis, characterized by both high sensitivity and specificity. Additionally, serodiagnosis assays based on chimeric proteins are easier to standardize and, thus, more reproducible. The above-mentioned advantages, together with the positive early indications presented here, suggest that further research on the use of chimeric proteins in the diagnosis of Lyme disease is worthwhile. Therefore, we propose these divalent chimeric proteins to be only the first phase of this research. Following these promising first results, we are already working on the construction of other divalent and trivalent chimeric proteins. Expanding our focus beyond just lipoproteins, this research will include a wider diversity *B. burgdorferi* s.l. antigens characterized by conserved amino acid sequence and immunogenicity. 

## 4. Materials and Methods

### 4.1. Serum Samples

Lyme disease serum samples were obtained from Department of Tropical Medicine and Epidemiology, Medical University of Gdańsk (Gdynia, Poland). IgG antibody levels were established using a commercial ELISA assay (*Borrelia* plus VlsE, Euroimmun, Lübeck, Germany). The presence of specific anti-*Borrelia* IgG antibodies was further confirmed using a commercial WB (EUROLINE WB *Borrelia*, Euroimmun, Lübeck, Germany). Twenty negative and 20 positive serum samples were used. All were obtained during routine borreliosis screening from the area of the Pomeranian Voivodeship (Poland). Anonymized information about each sample included only the date of collection and the titer of anti-*Borrelia* antibodies.

### 4.2. Amino Acid Sequence Analysis

Amino acid sequences of surface proteins from the five genospecies of *B. burgdorferi* s.l. (*B. afzelii*, *B. garinii*, *B. bavariensis*, *B. burgdorferi* s.s., and *B. spielmanii*) (Table 1) were aligned using Clustal X-2.1 software to identify the antigens with the most conserved sequences. The degree of conservation of the amino acid sequences was 88–99%, 79–97% and 77–100% for BmpA, BBK32 and BBA64, respectively. Next, conserved immunodominant regions were predicted using B Cell Epitope Prediction Tools (http://tools.immuneepitope.org/main/bcell/ accessed on 7 January 2021).

### 4.3. Construction of Recombinant Plasmids

Fragments of the *bmpA*, *bba64*, and *bbk32* genes were PCR-amplified from DNA of *B. burgdorferi* s.s. strain B31 (#35210DQ, ATCC, Manassas, VA, USA). PCR products contained complementary fragments incorporated into a sequence of primers (Table 2). In order to link two partially complementary DNA fragments into one gene encoding the chimeric protein (BmpA-BBA64 or BmpA-BBK32), a one-step PCR reaction was performed. The new DNA constructs were then used as a template in a standard PCR reaction. The final PCR products were digested with both *Bgl*II and *Xho*I and inserted into a pUET1 plasmid [60], so the chimeric proteins contain His-tags at both ends allowing for purification of the recombinant proteins by means of metal affinity chromatography. Figures S4 and S5 showing pUET1/BmpA-BBA64 and pUET1/BmpA-BBK32 maps are included in Supplementary Materials. The nucleotide sequences of the recombinant plasmids were confirmed by DNA sequencing (Genomed, Poland).

### 4.4. Expression and Purification of Chimeric Proteins 

*E. coli* BL21(DE3)pLysS transformed with either of the recombinant plasmids pUET1/BmpA-BBK32 or pUET1/BmpA-BBA64 was grown in TB broth supplemented with 100 μg/mL ampicillin and 34 μg/mL chloramphenicol to an optical density at λ = 600 nm of 0.4. Protein production was then induced with isopropyl-β-D-thiogalactopyranoside (IPTG) at a final concentration of 1 mM, and cells were incubated with vigorous shaking at 30°C for 18 h. Cells were then harvested by centrifugation. Proteins were purified in a one-step chromatography procedure by metal affinity chromatography with Ni^2+^ bound to an iminodiacetic acid-Sepharose column, in accordance with the manufacturer’s instructions (Novagen, Madison, WI, USA). Purity of the chimeric proteins was verified by 12% SDS–PAGE, quantified using Image Lab software (Bio-Rad, Hercules, CA, USA). After purification, the His-tags were not cleaved prior to immunoblot analysis. Protein concentration was measured with a Bradford Assay Kit according to the manufacturer’s instructions (Bio-Rad, Hercules, CA, USA) using bovine serum albumin (BSA) as a standard. 

### 4.5. Western Blot

For WB analysis, 7 µg of each recombinant protein was separated by 12% SDS-PAGE and then transferred onto nitrocellulose membrane. The membrane was blocked with 5% non-fat skim milk in Tris-buffered saline with 0.1% Tween 20 (TBS-T) for 1 h at room temperature with constant shaking. The membrane was then washed three times with TBS-T and incubated with human serum samples diluted at 1:200 for 1 h. The membrane was then washed again and treated for 1 h at room temperature with horseradish peroxidase-conjugated goat anti-human IgG antibodies (Jackson ImmunoResearch, Ely, UK), diluted at 1:100,000. After further washes, the reaction was developed by the addition of chemiluminescence peroxidase substrate (Immobilon Crescendo Western HRP substrate, Merck, Darmstadt, Germany), and results were visualized using Image Lab software (Bio-Rad, Hercules, CA, USA).

## Figures and Tables

**Table 1:** Accession numbers of BmpA, BBK32, and BBA64 protein amino acid sequences used in this study.

Protein	Genospecies	Accession no.
BmpA	*B. burgdorferi* B31 *B. burgdorferi* JD1 *B. burgdorferi* ZS7 *B. burgdorferi* N40 *B. burgdorferi* 156a	AAC66757.1 ADQ30753.1 ACK74429.1 ADQ29420.1 EEC22066.1
	*B. afzelii* Pko *B. afzelii* K78 *B. afzelii* HLJ01 *B. afzelii* Tom3107	ABH01648.1 AJY72370.1 AFU74676.1 WP_044052195.1
	*B. garinii* 20047 *B. garinii* NMJW1 *B. garinii* BgVir *B. garinii* PBr	AZA27810.1 AFT83711.1 AEW68720.1 WP_004791868.1
	*B. bavariensis* PBi	AAU07235.1
	*B. spielmanii* A14S	WP_006433486.1
BBK32	*B. burgdorferi* B31 *B. burgdorferi* N40 *B. burgdorferi* IA *B. burgdorferi* 156a *B. burgdorferi* PKa2 *B. burgdorferi* ZS7	AAC66134.1 WP_014540529.1 AAL84596.1 ACL33888.1 ACR57084.1 WP_012614929.1
	*B. afzelii* PKo *B. afzelii* K78 *B. afzelii* ACA-1 *B. afzelii* 570 *B. afzelii* A91	ACR57085.1 AJY72931.1 ACJ73236.1 AAL84590.1 AAL84589.1
	*B. garinii* 40 *B. garinii* PHei *B. garinii* TN *B. garinii* 50 *B. garinii* 46	AAL84593.1 ACO05729.1 ACR57087.1 AAL84595.1 AAL84594.1
	*B. bavariensis* PBi	ACR57086.1
	*B. spielmanii* A14S	WP_012666206.1
BBA64	*B. burgdorferi* B31 *B. burgdorferi* N40 *B. burgdorferi* JD1 *B. burgdorferi* 156a *B. burgdorferi* ZS7	AAC66255.2 ACS94806.1 ACS94870.1 ACL33793.1 ACK74216.1
	*B. afzelii* PKo *B. afzelii* K78 *B.* *afzelii* ACA-1	AEL70678.1 AJY72875.1 ACJ73562.1
	*B. garinii* BgVir *B. garinii* Far04 *B. garinii* PBr	AEW69221.1 ACL35142.1 ACL34799.1
	*B. bavariensis* PBi	AAT93822.1
	*B. spielmanii* A14S	ACN53346.1

**Table 2:** Oligonucleotide primers used for construction of chimeric proteins BmpA-BBA64 and BmpA-BBK32.

Primer Name	Primer Sequence	Comments
bmpABglII (Forward)	5’-GTGACAGATCTCGAATTTAAAATTGAGCTTC-3’	*Bgl*II site and fragment of *bmpA*
bmpA-bba64 (Reverse)	5’-GATAAAATTTGCCCAAGATTAATAAATTCTTTAAGAAAC-3’	Fragments of *bba64 *and *bmpA*
bmpA-bbba64 (Forward)	5’-GTTTCTTAAAGAATTTATTAATCTTGGGCAAATTTTATC-3’	Fragments of *bmpA* and *bba64*
bba64XhoI (Reverse)	5’-CATAACTCGAGCTGAATTGGAGCAAG-3’	*Xho*I site and fragment of *bba64*
bmpA-bbk32 (Reverse)	5’-GATATCGATTGCTTAATCTAATAAATTCTTTAAGAAACTTC-3’	Fragments of *bbk32 *and *bmpA*
bmpA-bbk32 (Forward)	5’-GAAGTTTCTTAAAGAATTTATTAGATTAAGCAATCGATATC-3’	Fragments of *bmpA* and *bbk32*
bbk32XhoI (Reverse)	5’-CATAACTCGAGGTACCAAACGCCATTC-3’	*Xho*I site and fragment of *bbk32*

## Data Availability

Publicly available datasets were analyzed in this study. This data can be found here: https://www.ncbi.nlm.nih.gov/protein accessed on 25 April 2021. The accession numbers are presented in Table 1.

## Supplementary Materials

The following are available online at https://www.mdpi.com/article/10.3390/pathogens10060767/s1. Figure S1: Multiple amino acid sequence alignment of the BmpA protein of *B. burgdorferi* s.l. deposited in NCBI; Figure S2: Multiple amino acid sequence alignment of the BBK32 protein of *B. burgdorferi* s.l. deposited in NCBI; Figure S3: Multiple amino acid sequence alignment of the BBA64 protein of *B. burgdorferi* s.l. deposited in NCBI; Table S1: The level of IgG antibodies and reactivity of chimeric proteins with individual sera; Figure S4: pUET1/BmpA-BBA64 map; Figure S5: pUET1/BmpA-BBK32 map.
