# Supplementary material for: Borrelia burgdorferi BmpA-BBK32 and BmpA-BBA64: New Recombinant Chimeric Proteins with Potential Diagnostic Value
Source: Pathogens. 2021 Jun 18;10(6):767. doi: 10.3390/pathogens10060767 (PMC8234703; doi:10.3390/pathogens10060767)
Supplement: Supplementary file 1 [file pathogens-10-00767-s001.zip › pathogens-1256786-supplementary.pdf]

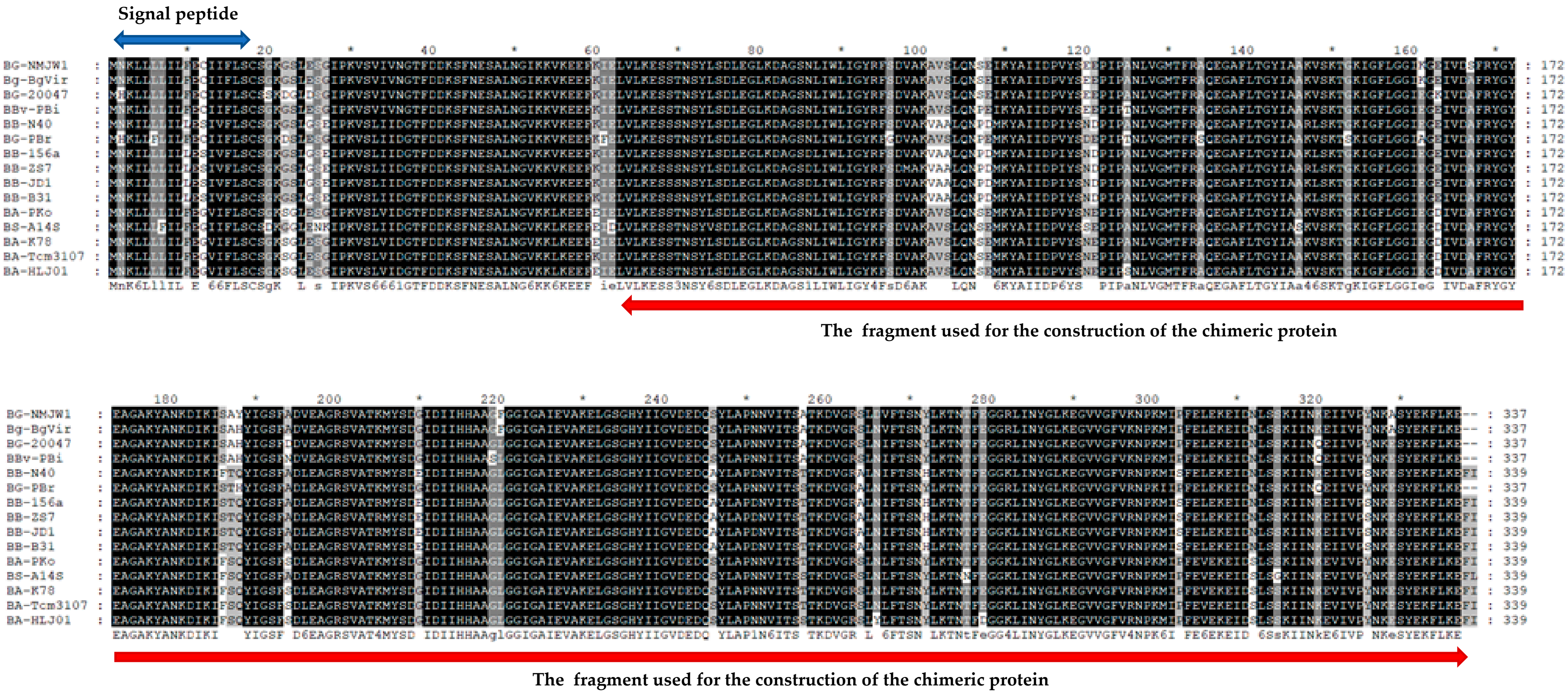

Figure S1. Multiple amino acid sequence alignment of the BmpA protein of *B. burgdorferi* s.l. deposited in NCBI

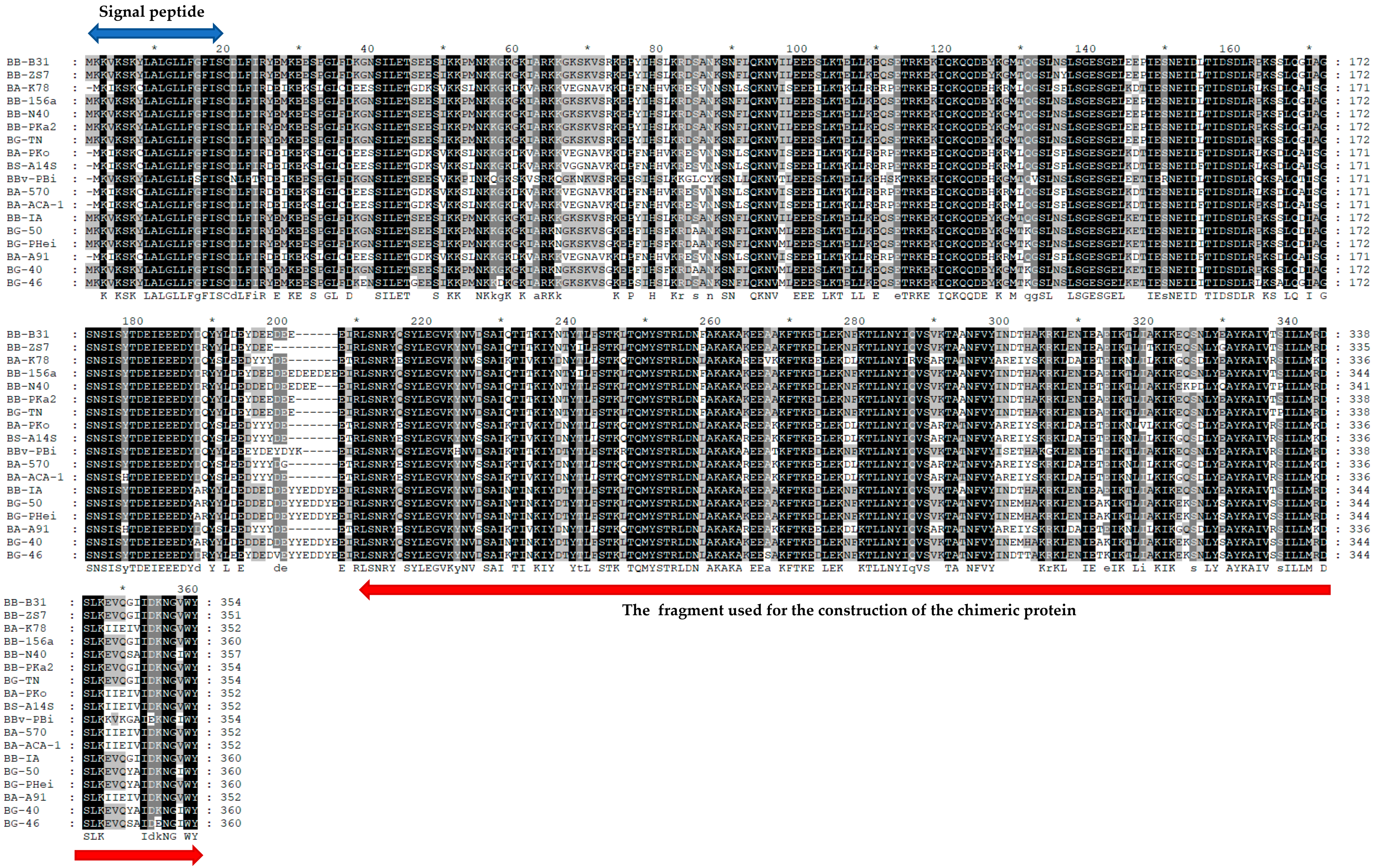

Figure S2. Multiple amino acid sequence alignment of the BBK32 protein of *B. burgdorferi* s.l. deposited in NCBI

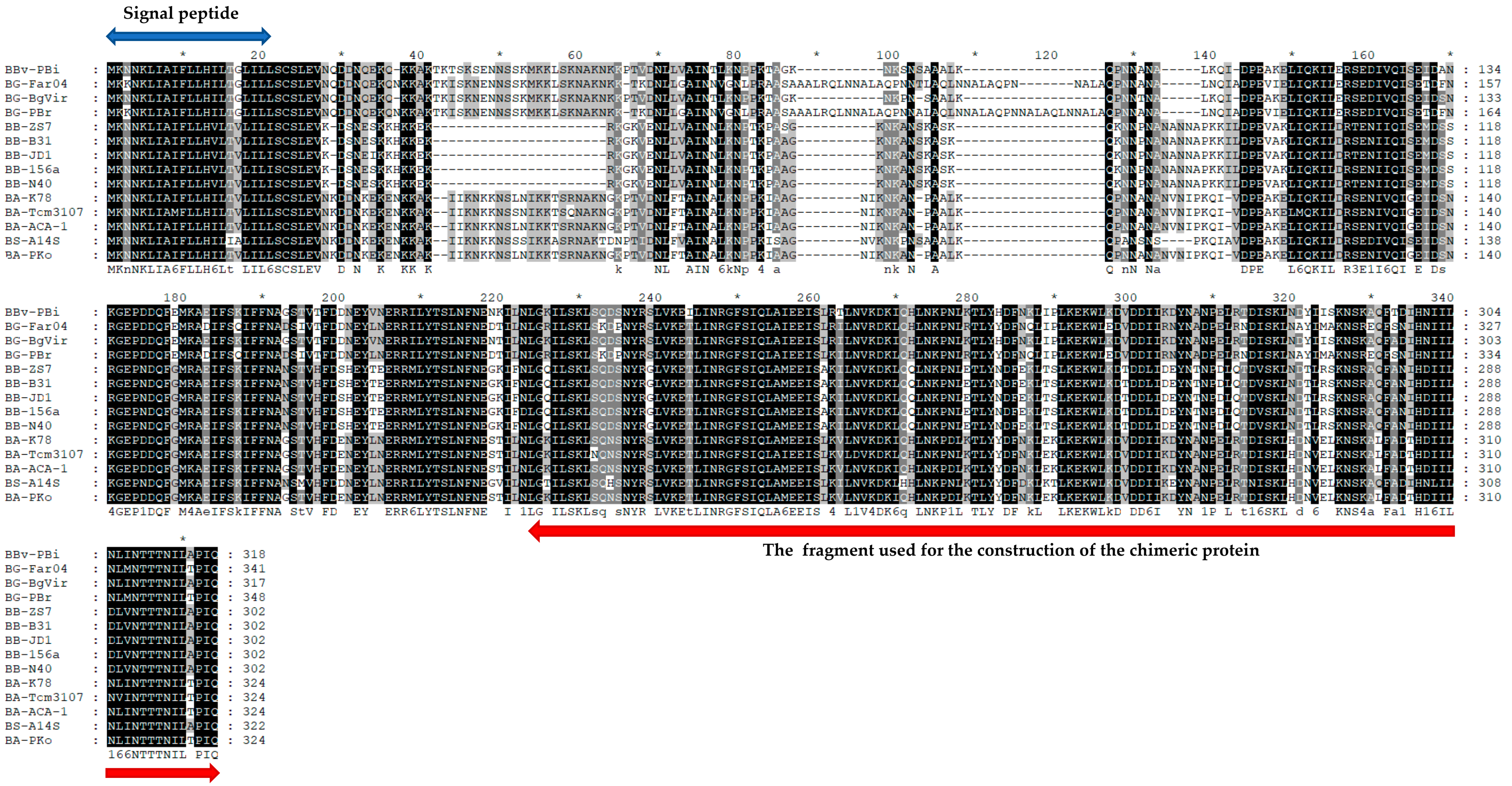

Figure S3. Multiple amino acid sequence alignment of the BBA64 protein of *B. burgdorferi* s.l. deposited in NCBI

Table S1. The level of IgG antibodies and reactivity of chimeric proteins with individual sera (bold sera for which the results are shown in Figure 1.)

**BmpA-BBA64**					**BmpA-BBK32**				
**Positive sera**			**Negative sera**		**Positive sera**			**Negative sera**	
**No.**	**IgG level**	**Results**	**No.**	**Results**	**No.**	**IgG level**	**Results**	**No.**	**Results**
1	35	Reactive	1	Non reactive	1	35	Reactive	1	Non reactive
2	39	Reactive	2	Non reactive	2	39	Reactive	2	Non reactive
3	42	Reactive	3	No reactive	3	42	Reactive	3	No reactive
4	48	Reactive	4	Non reactive	4	48	Reactive	4	Non reactive
5	51	Reactive	5	Non reactive	5	51	Reactive	5	Non reactive
6	56	Reactive	6	Non reactive	6	56	Reactive	6	Non reactive
**7**	**59**	**Reactive**	7	Non reactive	**7**	**59**	**Reactive**	7	Non reactive
8	65	Reactive	8	Non reactive	8	65	Reactive	8	Non reactive
9	71	Reactive	9	Non reactive	9	71	Reactive	9	Non reactive
10	77	Reactive	10	Non reactive	10	77	Reactive	10	Non reactive
11	80	Reactive	11	Non reactive	11	80	Reactive	11	Non reactive
12	86	Reactive	12	Non reactive	12	86	Reactive	12	Non reactive
13	94	Reactive	13	Non reactive	13	94	Reactive	13	Non reactive
**14**	**98**	**Reactive**	14	Non reactive	**14**	**98**	**Reactive**	14	Non reactive
15	111	Reactive	15	Non reactive	15	111	Reactive	15	Non reactive
16	137	Reactive	16	Non reactive	16	137	Reactive	16	Non reactive
17	149	Reactive	17	Non reactive	17	149	Reactive	17	Non reactive
18	178	Reactive	18	Non reactive	18	178	Reactive	18	Non reactive
19	195	Reactive	19	Non reactive	19	195	Reactive	19	Non reactive
**20**	**203**	**Reactive**	20	Non reactive	**20**	**203**	**Reactive**	20	Non reactive
**Summary**		**20/20**		**0/20**	**Summary**		**20/20**		**0/20**

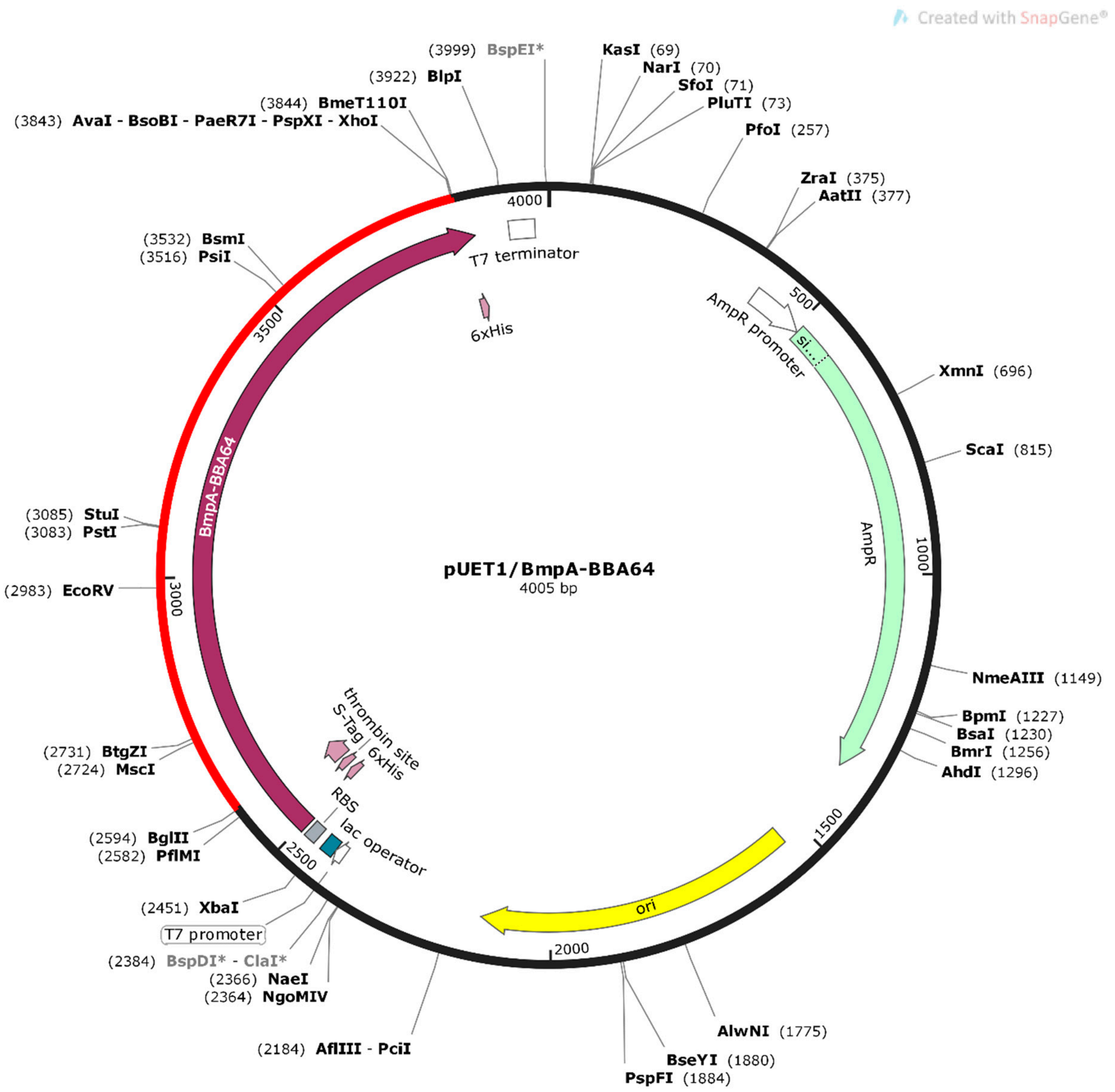

Figure S4. pUET1/BmpA-BBA64 map

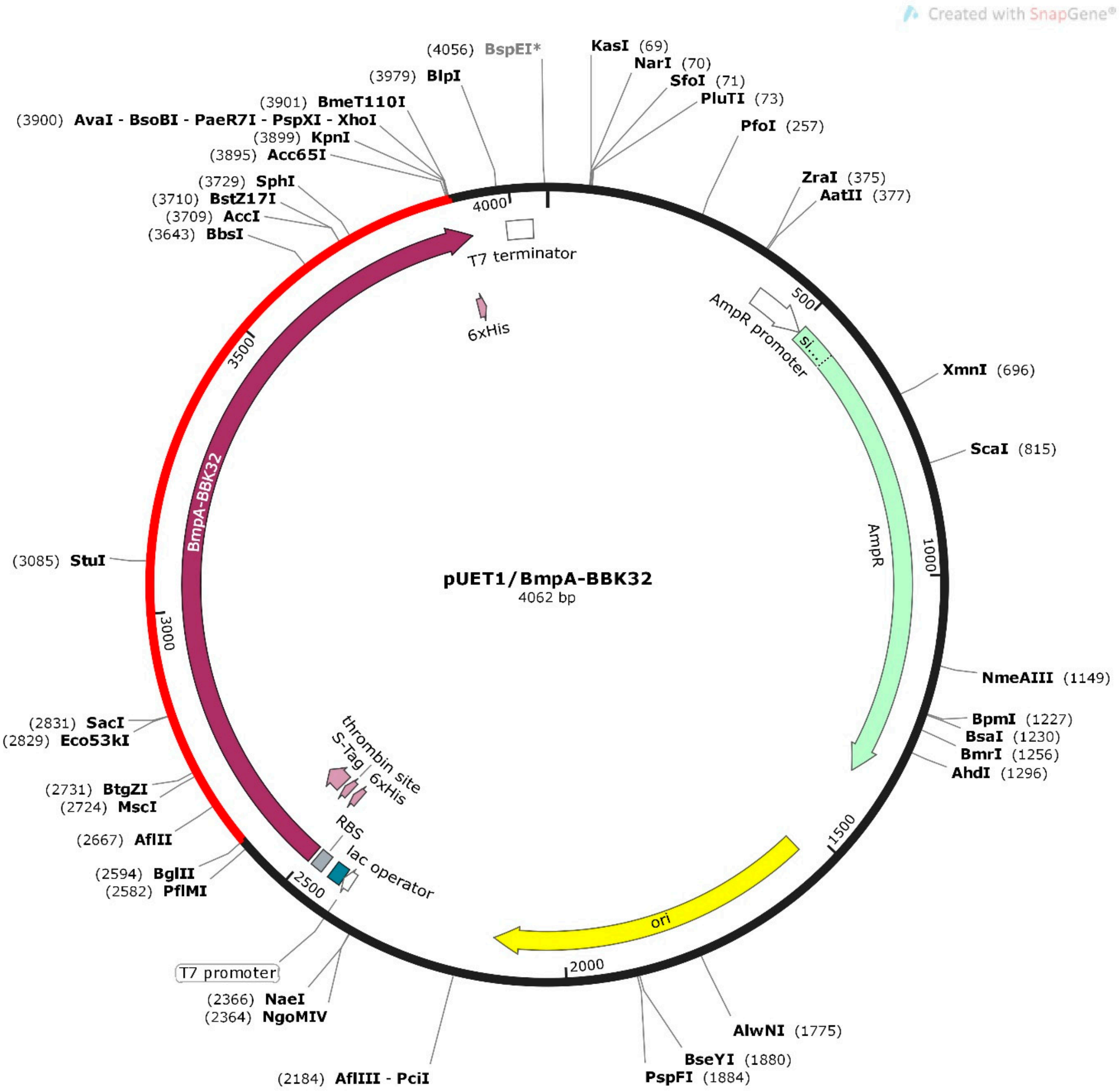

Figure S5. pUET1/BmpA-BBK32 map
